# Cefazolin and imipenem enhance AmpC expression and resistance in NagZ-dependent manner in *Enterobacter cloacae* complex

**DOI:** 10.1186/s12866-022-02707-7

**Published:** 2022-11-29

**Authors:** Xianggui Yang, Zhenguo Wang, Mingquan Liu, Xuejing Yu, Yuanxiu Zhong, Fuying Wang, Ying Xu

**Affiliations:** 1 Department of Laboratory Medicine, Clinical Medical College and the First Affiliated Hospital of Chengdu Medical College, Chengdu, Sichuan China; 2 Department of Stomatology, Clinical Medical College and the First Affiliated Hospital of Chengdu Medical College, Chengdu, Sichuan China; 3 Department of Internal Medicine, Division of Cardiology, University of Texas Southwestern Medical Center, Dallas, TX USA; 4 Department of Biotechnology, Chengdu Medical College, Chengdu, Sichuan China

**Keywords:** Cefazolin, Imipenem, AmpC, NagZ, *Enterobacter cloacae* complex

## Abstract

**Background:**

*Enterobacter cloacae* complex (ECC) is a common opportunistic pathogen and is responsible for causing various infections in humans. Owing to its inducible chromosomal AmpC β-lactamase (AmpC), ECC is inherently resistant to the 1st- and 2nd- generation cephalosporins. However, whether β-lactams antibiotics enhance ECC resistance remains unclear.

**Results:**

In this study, we found that subinhibitory concentrations (SICs) of cefazolin (CFZ) and imipenem (IMP) can advance the expression of AmpC and enhance its resistance towards β-lactams through NagZ in *Enterobacter cloacae* (EC). Further, AmpC manifested a substantial upregulation in EC in response to SICs of CFZ and IMP. In *nagZ* knockout EC (Δ*nagZ*), the resistance to β-lactam antibiotics was rather weakened and the effect of CFZ and IMP on AmpC induction was completely abrogated. NagZ ectopic expression can rescue the induction effects of CFZ and IMP on AmpC and increase Δ*nagZ* resistance. More importantly, CFZ and IMP have the potential to induce the expression of AmpR's target genes in a NagZ-dependent manner.

**Conclusions:**

Our findings suggest that NagZ is a critical determinant for CFZ and IMP to promote AmpC expression and resistance and that CFZ and IMP should be used with caution since they may aggravate ECC resistance. At the same time, this study further improves our understanding of resistance mechanisms in ECC.

**Supplementary Information:**

The online version contains supplementary material available at 10.1186/s12866-022-02707-7.

## Background

*Enterobacter cloacae*, *Enterobacter ludwigii, Enterobacter hormaechei, Enterobacter nimipressuralis, Enterobacter asburiae,* and *Enterobacter kobei* combinedly referred to as *Enterobacter cloacae* complex (ECC). They are grouped within *Enterobacter* and have a wide range of prevalence [1, 2]. Among ECC, *Enterobacter cloacae* is isolated frequently from clinical specimens obtained from humans as well as medical devices, and it has gained clinical significance in intensive care patients, particularly those on mechanical ventilation [1]. Owing to the antibiotic-resistance characteristics of microorganisms, ECC has been a point of focus of quite a large number of publications [2–4]. ECC is inherently resistant to amoxicillin, ampicillin, 1st- and 2nd-generation of cephalosporin, and cefoxitin on account of the generation of inducible chromosomes AmpC β-lactamase (AmpC) [5].

NagZ, also known as the β‐N‐ acetylglucosaminidase, is a crucial enzyme that takes part in peptidoglycan recycling and has the potential to cleave GlcNAc‐1,6‐anhydroMurNAc‐peptides into N-acetyl-β-d-glucosamine and 1,6‐anhydroMurNAc‐peptides (anhMurNAc) [6, 7]. NagZ inactivation has been demonstrated to reduce resistance to β-lactam antibiotics in *Pseudomonas aeruginosa* [8, 9]*, Stenotrophomonas maltophilia* [10], and *Yersinia enterocolitica* [11]. Moreover, within *Neisseria gonorrhoeae*, NagZ can also regulate the accumulation of biofilm [12]. In *Pseudomonas aeruginosa*, anhMurNAc enhances AmpC expression by activating AmpR (a global transcriptional factor responsible for regulating hundreds of genes including *ampC*) [9, 13]. Despite these promising findings, the precise regulatory mechanism in ECC is still unclear.

*ampC* gene is frequently found on the chromosomes of non-fermenting bacteria such as *Pseudomonas aeruginosa* and some *Enterobacteriaceae* such as *Enterobacter cloacae*, *Citrobacter freundii* and *Enterobacter asburiae* [5]. *ampC* overexpression renders these pathogens resistant to penicillin, the first and second-generation cephalosporins, and β-lactam/β-lactamase inhibitors [14], as well as carbapenems, especially with porin loss [3, 15]. The overproduction of AmpC is the major cause of ECC's cephalosporin resistance [16]. It is reported that cefoxitin and cefotaxime can induce AmpC expression [17], which consequently results in antibiotic treatment failure, but the potential inducible mechanism is not unclear.

The main objective of the current study is to investigate whether other β-lactam antibiotics (except cefoxitin and cefotaxime) could induce AmpC expression and to explore the induction mechanism. As discovered in this study, NagZ is a key intermediate regulator in the process of AmpC induction expression by CFZ and IMP in ECC. Our findings also suggest that CFZ and IMP should be used with caution, as they have the potential to exacerbate ECC resistance and make therapy more challenging.

## Results and discussion

### SICs of CFZ and IMP improve the expression of AmpC and enhance resistance to β-lactam in EC clinical isolate

**Fig. 1:**
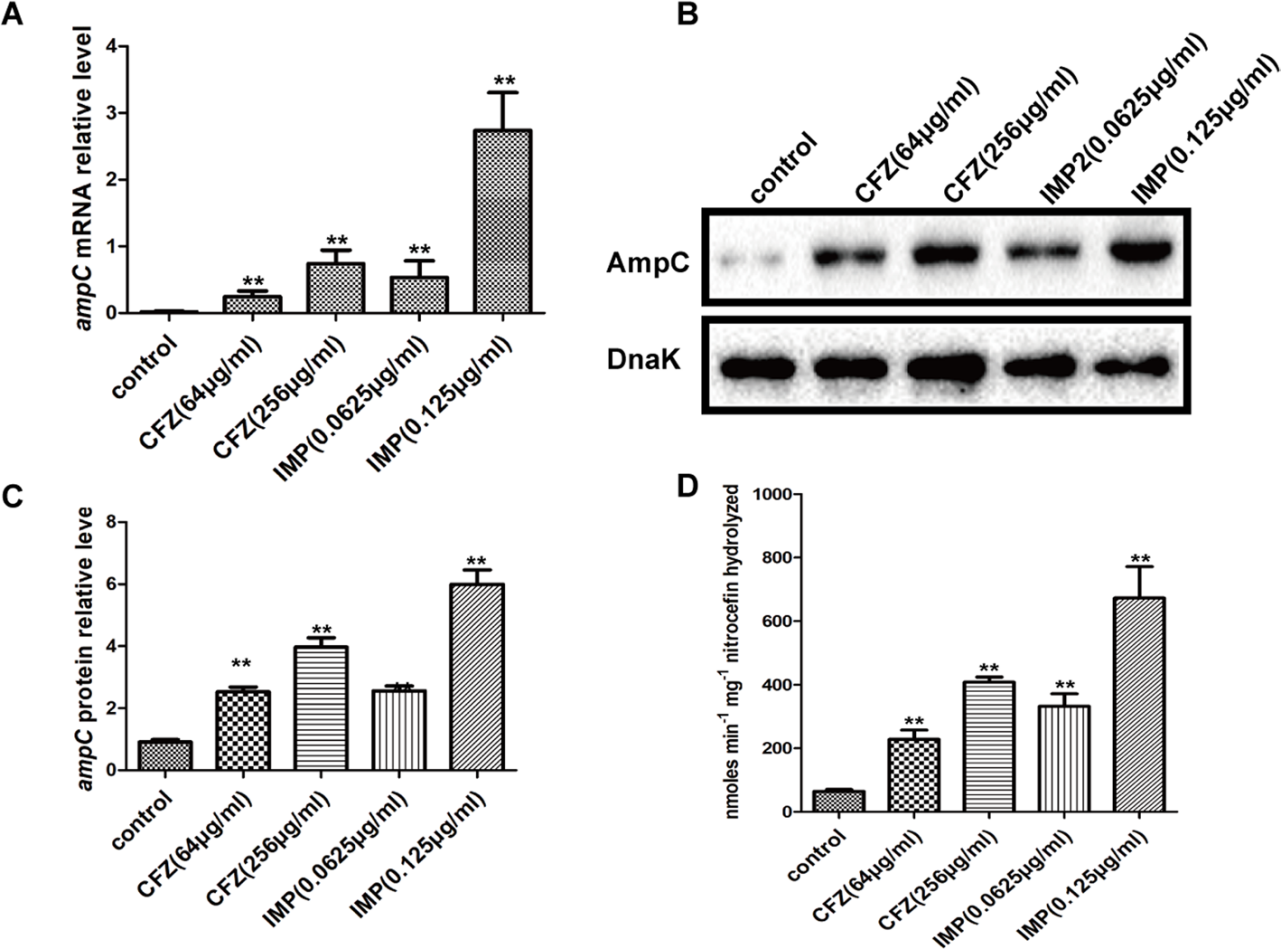
Effects of SICs of CFZ and IMP on AmpC expression and activity in EC clinical isolate. **A** Analysis of the *ampC* mRNA expression levels was made using RT-qPCR in EC clinical isolate treated with SICs of CFZ (64 μg/ml and 256 μg/ml) and IMP (0.0625 μg/ml and 0.125 μg/ml). **B** Western blot (cropped blots) analysis of *ampC* protein expression in EC clinical isolate treated with SICs of CFZ and IMP. **C** Quantitative analysis of the western blot results (**B**) with image J software, DnaK was employed as an internal control. **D** Determination of the effects of SICs of CFZ and IMP on AmpC activity by nitrocefin hydrolysis assay. ** *P* < 0.01 suggests the high statistical significance

The growing resistance to β-lactam antibiotics, the commonly used antibiotics for treating gram-negative bacterial infections, is a major concern in clinical practice [18]. β-lactamase is an enzyme that cleaves the cyclic amide component of β-lactams, thus rendering them inactive [19]. One type of lactamases that poses serious challenges to the antibiotic treatment is inducible chromosomal AmpC β-lactamase (AmpC), which has broad-spectrum activity against β-lactams [20]. It was reported that cefoxitin and low concentration cefotaxime could improve AmpC expression [17]. To investigate whether other antibiotics can elicit the expression of AmpC, firstly, various antibiotics (including quinolones, β- lactams, and aminoglycosides) were tested for their minimum inhibitory concentration (MIC) against EC clinical isolate following the guideline outlined by the Clinical Laboratory Standard Institute (CLSI) [21]. The results are shown in Table S1. Later, the western blot assay was employed for determining whether or not the subinhibitory concentration (SIC, ≤ 1/4 MIC) of antibiotics induce AmpC expression. The findings (Fig. S1) demonstrate that CFZ, IMP, and cefoxitin have a strong induction impact on AmpC, and ceftriaxone, cefotaxime, ceftazidime, and cefepime have a modest induction effect, while other antibiotics, such as aminoglycosides and quinolones, showed no discernible effect on AmpC. Next, we explored the effect of induction of different concentrations CFZ and IMP on AmpC expression at mRNA level by reverse transcription-quantitative polymerase chain reaction (RT-qPCR) and protein level by western blot, respectively. According to our findings (Fig. 1 A-C), CFZ and IMP have a dosage impact on AmpC induction. Finally, the AmpC generated by CFZ and IMP was tested using a nitrocefin hydrolysis assay to see if it had good β-lactamase activity. The results show that CFZ and IMP increase AmpC β-lactamase activity in a dose-dependent manner when compared to the control group (Fig. 1D).

The high expression of AmpC has been reported as one of the main causes for gram-negative bacilli resistance to β-lactams [22, 23], so next, the role of CFZ and IMP in resistance was investigated. The inhibition zone and MICs of aztreonam (ATM), ceftriaxone (CRO), ceftazidime (CAZ), piperacillin (PIP), piperacillin-tazobactam (TZP), and cefoperazone-sulbactam (SCF) against EC clinical isolate treated with or without SICs of CFZ and IMP were estimated by broth microdilution method and disk diffusion technique (Kirby-Bauer method) following the CLSI guideline [21], as indicated in Table 1 and Fig. S2A, inhibition zones of those antibiotics against EC treated with SICs of CFZ and IMP decreased as compared with the control group, and the MICs of TZP, PIP, CRO, SCF, CAZ, and ATM manifested a substantial increase by SICs of CFZ and IMP.

**Table 1:** MICs for the ECC clinical isolate treated with CFZ and IMP

Antibiotics	MIC(μg/ml)				
	**control**	**CFZ(64 μg/ml)**	**CFZ(256 μg/ml)**	**IMP(0.0625 μg/ml)**	**IMP(0.125 μg/ml)**
PIP	64	128	512	512	1024
TZP	32	128	256	512	1024
ATM	32	64	256	256	512
CRO	16	32	64	256	512
CAZ	16	64	128	128	256
SCF	4	8	32	32	128

*Abbreviations*: *MIC* Minimum inhibitory concentration, *CFZ* Cefazolin, *IMP* Imipenem, *PIP* Piperacillin, *TZP* Piperacillin-tazobactam, *ATM* Aztreonam, *CRO* Ceftriaxone, *CAZ* Ceftazidime; and *SCF* Cefoperazone-sulbactam

CFZ and IMP are broad-spectrum β-lactam antibiotics used against bacteria, including aerobes and anaerobes. Furthermore, CFZ is a commonly used antibiotic for empiric therapy and postoperative infection prevention [24, 25]. Since CFZ and IMP may exacerbate EC resistance by inducing AmpC expression, our findings imply that they should be used with caution.

### The effects of induction of CFZ and IMP on AmpC expression and resistance were abrogated in ΔnagZ

**Fig. 2:**
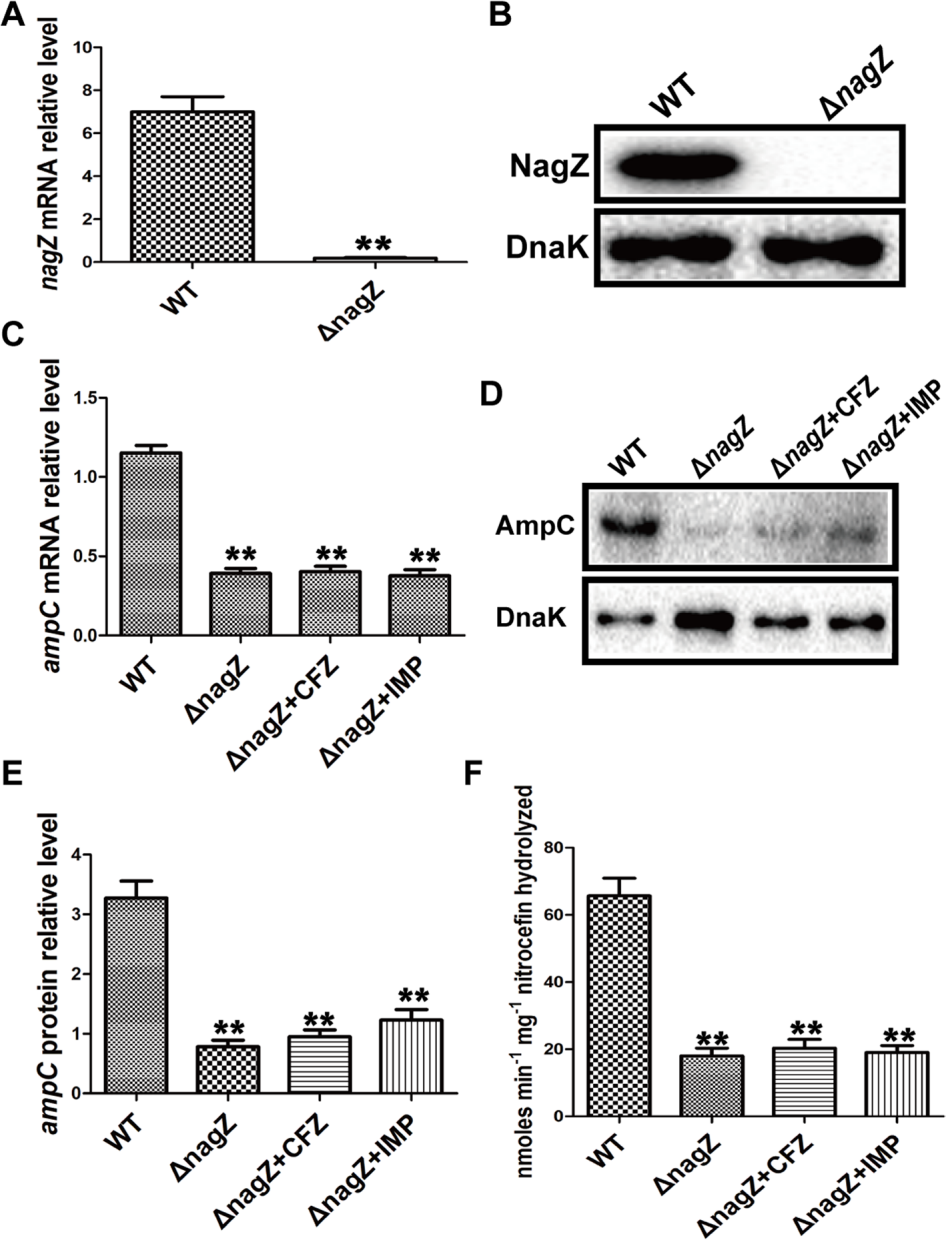
The role of NagZ in *ampC* expression and resistance in EC. RT-qPCR (**A**) and cropped Western Blot (**B**) verified that *nagZ*-knockout EC clinical isolate (Δ*nagZ*) was successfully constructed. **C**-**D** RT-qPCR and Western Blot (cropped blots) were employed for the determination of the role of NagZ in the expression of AmpC induced by subinhibitory concentration CFZ (256 μg/ml) and IMP (0.125 μg/ml) in EC clinical isolate (WT), Δ*nagZ*, Δ*nagZ* treated with CFZ (Δ*nagZ* + CFZ) and Δ*nagZ* treated with IMP (Δ*nagZ* + IMP). **E** Western blot Quantitative analysis (**D**), The internal control employed was DnaK. **F** Nitrocefin hydrolysis assay in WT, Δ*nagZ*, Δ*nagZ* + CFZ, and Δ*nagZ* + IMP was employed to examine the role of NagZ in AmpC β-lactamase activity. ** *P* < 0.01 indicates high statistical significance

As described above, some β-lactams can induce AmpC expression, but the underlying regulatory mechanism, which is intricately involved in peptidoglycan recycling, remains unclear [26, 27]. NagZ, existing in Gram-negative bacteria and involved in the peptidoglycan recycling pathway, is identified as an exo-N-acetyl-β-glucosaminidase. In some Gram-negative bacteria, NagZ inactivation has been reported to arrest and reverse the resistance to β-lactam antibiotics [28, 29]. As shown in Fig. S2A and Table 1, our data indicate the SICs of CFZ and IMP have the potential to aggravate the resistance of EC, so we speculated that NagZ may have an indispensable part in promoting resistance of EC. In an attempt to confirm the hypothesis, the knockout model of gene *nagZ* (Δ*nagZ*) was constructed by employing homologous recombination technology in EC clinical isolate (WT). As shown by RT-qPCR (Fig. 2A) and western blot (Fig. 2B), the *nagZ* gene was effectively knocked out. Next, we determined the effects of SICs of CFZ and IMP on the expression and activity of AmpC in Δ*nagZ*. The results, as shown in Fig. 2C-F, depict that the expression of AmpC was significantly downregulated, and the induction of AmpC by SICs of CFZ and IMP was completely abolished in Δ*nagZ* (Fig. 2C-E). At the same time, knocking down *nagZ* reduced AmpC's rising β-lactamase activity produced by SICs of CFZ and IMP (Fig. 2F). Furthermore, antibiotic susceptibility tests revealed that EC resistance had been considerably reduced in Δ*nagZ*, and that CFZ and IMP had little impact on resistance in Δ*nagZ* (Table 2 and Fig. S2B). Thus, our results indicated that the induction of AmpC by CFZ and IMP was dependent on NagZ.

**Table 2:** The effect of *nagZ* on MICs for six anbiotics

Antibiotics	MIC(μg/ml)						
	**WT**	**Δ*nagZ***	**Δ*nagZ* + CFZ** **(4 μg/ml)**	**Δ*nagZ* + IMP** **(0.0625 μg/ml)**	**Δ*nagZ* + NagZ**	**Δ*nagZ* + NagZ + ** **CFZ(256 μg/ml)**	**Δ*nagZ* + NagZ + ** **IMP(0.125 μg/ml)**
PIP	64	2	2	1	32	256	512
TZP	32	2	1	0.5	32	128	512
ATM	32	1	1	0.5	16	256	512
CRO	16	0.5	0.5	0.25	32	64	1024
CAZ	16	0.5	0.5	0.25	16	64	128
SCF	4	0.25	0.125	0.0625	4	64	64

*Abbreviations*: *ΔnagZ nagZ*-knockout *Enterobacter cloacae* complex, *ΔnagZ* + *CFZ* Δ*nagZ* treated with CFZ, *ΔnagZ* + *IMP* Δ*nagZ* treated with IMP, *ΔnagZ* + *NagZ* + *CFZ* Δ*nagZ* + NagZ treated with CFZ, *ΔnagZ* + *NagZ* + *IMP* Δ*nagZ* + NagZ treated with IMP

François Guérin and his colleagues found that cefotaxime induced the expression of AmpC in a NagZ-dependent manner, while the induction of AmpC by cefoxitin was independent of NagZ [17]. As we know, CFZ and cefotaxime are cephalosporin, and cefoxitin belongs to cephamycin. We suspect that cephalosporin and carbapenem are dependent on NagZ for the induction of AmpC, while cephamycin is independent of NagZ. Therefore, in the follow-up work, we will explore the induction mechanism of AmpC from the perspective of β-lactam's molecular structure.

### Ectopic expression of NagZ rescues induction effect of CFZ and IMP on AmpC expression and resistance in ΔnagZ

**Fig. 3:**
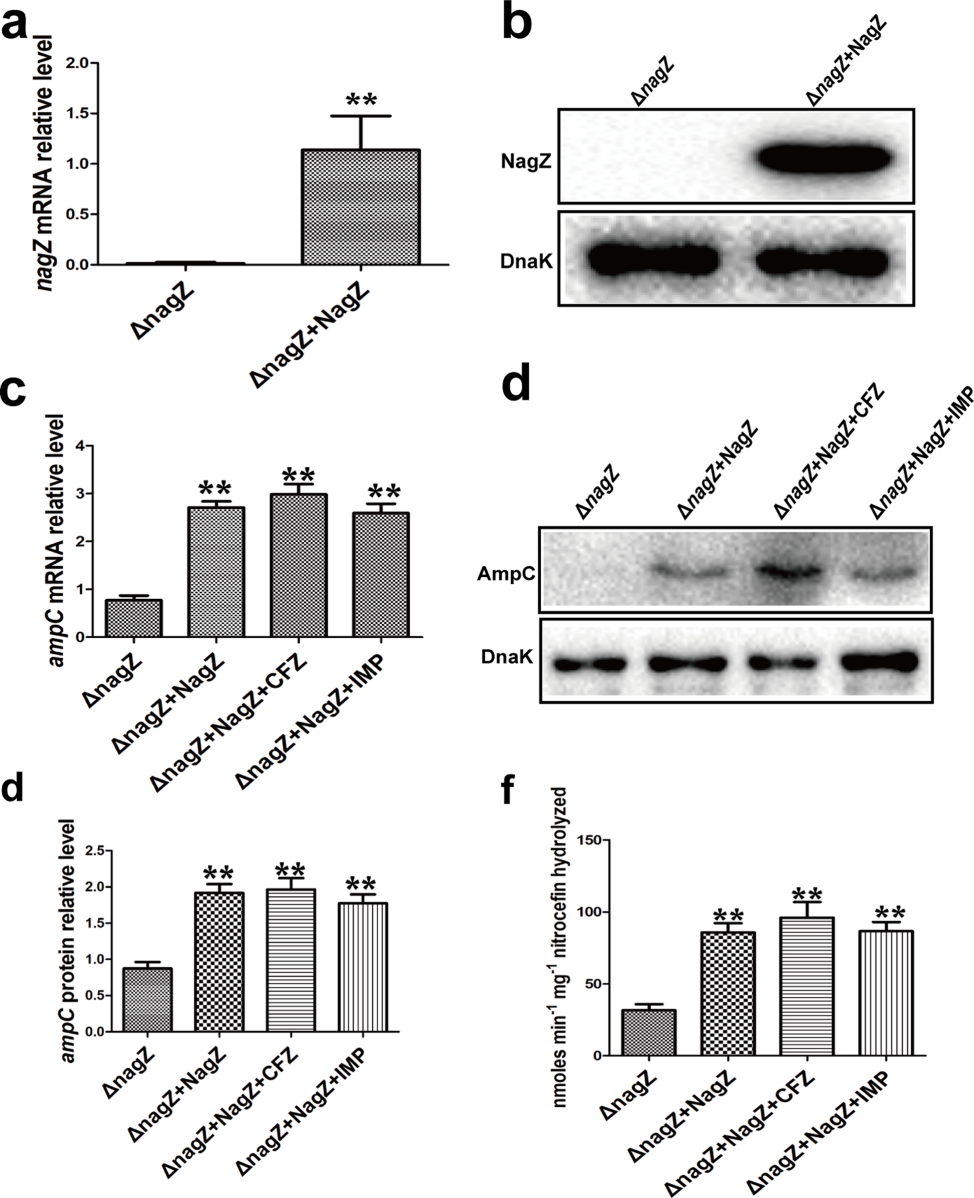
NagZ complementation can rescue induction of AmpC and resistance by subinhibitory concentration CFZ (256 μg/ml) and IMP (0.125 μg/ml) in Δ*nagZ*. RT-qPCR (**A**) and cropped western blot (**B**) verified that the NagZ complementation model was successfully generated. **C** mRNA expressions of *ampC* were identified using RT-qPCR in Δ*nagZ*, Δ*nagZ* complemented with NagZ (Δ*nagZ* + NagZ), Δ*nagZ* + NagZ treated with CFZ (Δ*nagZ* + NagZ + CFZ) and Δ*nagZ* + NagZ treated with IMP (Δ*nagZ* + NagZ + IMP). **D** Western blot (cropped blots) was employed for the determination of *ampC* protein expressions in Δ*nagZ*, Δ*nagZ* + NagZ, Δ*nagZ* + NagZ + CFZ, and Δ*nagZ* + NagZ + IMP strains. **E** Western blot Quantitative analysis (**D**), and the internal control employed was DnaK. **F** Nitrocefin hydrolysis assay in *nagZ*, Δ*nagZ* + NagZ, Δ*nagZ* + NagZ + CFZ and Δ*nagZ* + NagZ + IMP was used for the analysis of AmpC β-lactamase activity. ** *P* < 0.01 indicates high statistical significance

To investigate whether NagZ complementation rescues the expression of *ampC* and enhances resistance in Δ*nagZ* treated with or without SICs of CFZ and IMP, the *nagZ* coding sequence (CDS) was cloned into the vector of pBAD33cm-rp4 (pBAD33-*nagZ*, *nagZ* overexpression vector). Later, the pBAD33-*nagZ* and the vector of pBAD33cm-rp4 (pBAD33, as control vector) were transformed into Δ*nagZ* by electroporator. RT-qPCR and western blot analyses were employed for detecting the availability of the pBAD33-*nagZ* vector (Fig. 3A, B). Next, the *ampC* expression was ascertained using western blot and RT-qPCR, the results showed that *ampC* levels of mRNA (Fig. 3C) and protein (Fig. 3D-E) were rescued by NagZ complementation in Δ*nagZ* in response to SICs of CFZ and IMP. Furthermore, NagZ was investigated in terms of its influence on the AmpC β-lactamase activity, and the result indicates that reduced activity of β- lactamase resulting from the elimination of *nagZ* was reversed by NagZ complementing in Δ*nagZ* (Fig. 3F). Additional confirmation of the significance of NagZ in resistance of Δ*nagZ* was carried out by measuring the inhibition zones and MICs of TZP, PIP, CRO, ATM, CAZ, and SCF. The findings demonstrate that NagZ overexpression may greatly reduce the inhibition zone and that SICs of CFZ or IMP can further reduce the inhibition zone (Fig. S2C). Consistent with the inhibition zone, NagZ complementation and SICs of CFZ or IMP can evidently increase the MICs (Table 2).

Briefly, the complementation of NagZ has the potential to rescue β-lactams resistance and the inducible effects of CFZ and IMP to AmpC in the *nagZ* knockout model. The data further indicate that CFZ and IMP enhance AmpC expression and resistance in a NagZ-dependent manner in EC clinical isolate.

### CFZ and IMP promote AmpC expression through the NagZ-AmpR-AmpC pathway

**Fig. 4:**
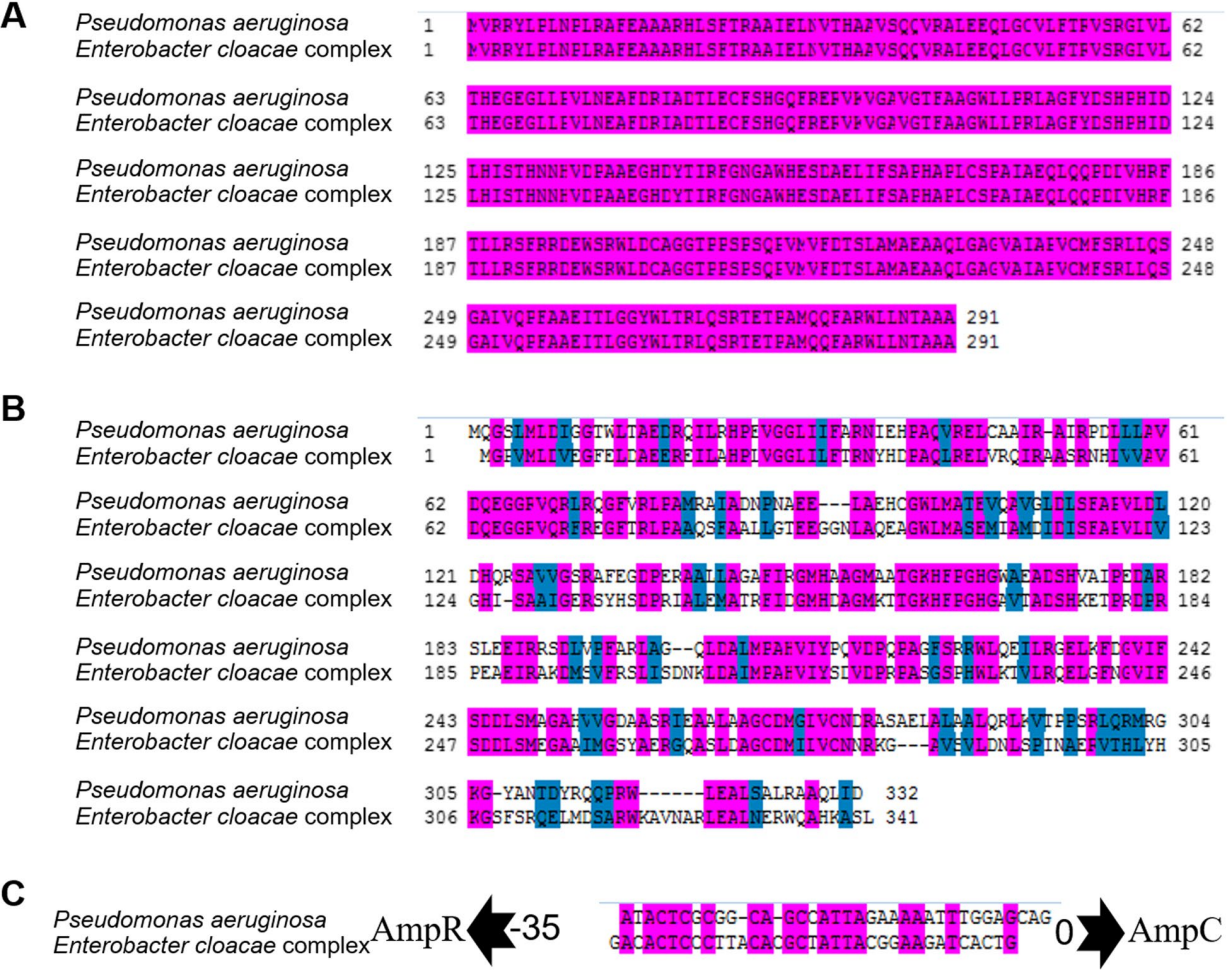
NagZ and AmpR sequence conservative analysis among *Enterobacter cloacae* and *Pseudomonas aeruginosa.* NagZ (**A**) and AmpR (**B**) amino acid sequence alignment among *Pseudomonas aeruginosa* and *Enterobacter cloacae*, the identical sequences are indicated using hot-pink, those marked by dark blue means the same class of amino acids with respect to their structure or function and marked by white means different types of amino acids. **C** The transcription binding region nucleotide sequence alignment of AmpC (about -35 bp) among *Pseudomonas aeruginosa* and *Enterobacter cloacae*, the conservative sequences are marked by hot-pink, the nucleotide sequences with differences between *Enterobacter cloacae* and *Pseudomonas aeruginosa* marked in white

**Fig. 5:**
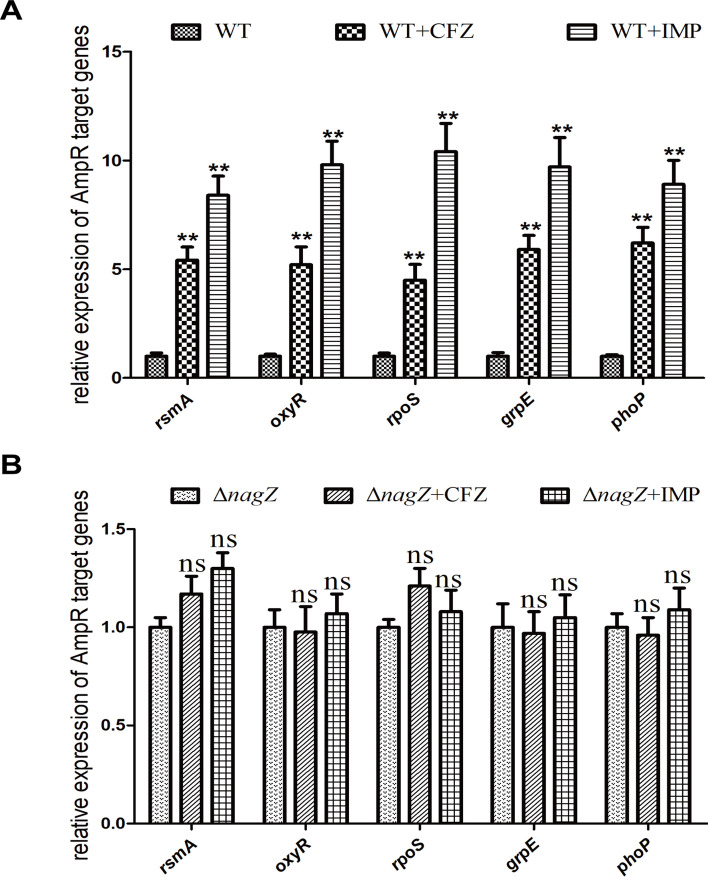
Influence of NagZ upon AmpR target genes expression, including *rsmA, oxyR, grpE, rpoS,* and *phoP*. mRNA expression of *rsmA, rpoS, oxyR, grpE,* and *phoP* was determined by RT-qPCR in EC (**A**) and Δ*nagZ* (**B**) treated with or without CFZ and IMP. ** *P* < 0.01 indicates high statistical significance

Peptides from peptidoglycan degradation are transported by AmpG permease into the cytoplasm. In the cytoplasm, GlcNAc‐1,6‐anhydroMurNAc‐peptides detach GlcNAc moiety with the help of NagZ and forms 1,6‐anhydroMurNAc‐peptides (anhMurNAc) [30]. Under normal physiological growth, AmpD cleaves anhMurNAc to generate free peptides and then synthesizes UDP-pentapeptides, which suppresses AmpR activity and represses AmpC transcription [5, 26, 31]. However, in the presence of inducers such as β-lactams, AmpD cannot cleave the high anhMurNAc concentration effectively. The accumulating anhMurNAc activates AmpR and increases AmpC transcription, which is also the main mechanistic step responsible for developing resistance to most β -lactams in *Pseudomonas aeruginosa* [30, 32]. Besides, several studies have shown that AmpR regulates the expression of a multitude of genes and is thus a global transcription factor (the genes regulated by AmpR include *oxyR, rsmA, rpoS, phoP,* and *grpE*) in *Pseudomonas aeruginosa* [32, 33]. Therefore, we hypothesize that, like *Pseudomonas aeruginosa*, there is a pathway in *Enterobacter cloacae* complex and that the induction of AmpC by SICs of CFZ and IMP is AmpR dependent. To confirm our hypothesis, *Pseudomonas aeruginosa* and *Enterobacter cloacae* were both analyzed for their NagZ and AmpR protein sequence conservations. The AmpR sequences of the two species were identified (Fig. 4A) and the conservation of the NagZ sequence was as high as 67% (Fig. 4B). In addition, a high homology was observed in the -35 bp-0 bp region (generally considered as transcriptional parameter zone of binding) for *ampC* between *Pseudomonas aeruginosa* and *Enterobacter cloacae* (Fig. 4C). The highly conserved sequence indicates that these genes function similar roles in vivo. Therefore, we next confirmed if the induction of CFZ and IMP to AmpC is dependent on NagZ-mediated AmpR activation. The effect of CFZ and IMP upon the expression of AmpR target genes in wild type EC and Δ*nagZ* was measured. The results imply that CFZ and IMP are able to promote the AmpR target genes expression (for instance *oxyR, rsmA, grpE, rpoS,* and *phoP*) in wild type EC (Fig. 5A), while in the Δ*nagZ* strain, CFZ and IMP did not affect the expressions of AmpR target genes (Fig. 5B).

Here, CFZ and IMP have been confirmed to promote AmpC through the NagZ-AmpR pathway. However, it is unclear how NagZ affects AmpR's transcriptional activity (for example, whether NagZ or its hydrolysate anhMurNAc can cause AmpR's conformational change, which is known to activate AmpR's transcriptional activity). Therefore, for future studies, we will collaborate with scientists in the field of protein structure to investigate the effect of NagZ and its hydrolysate anhMurNAc on the structure of AmpR. In addition, the data of only one clinical strain were applied in this study. In fact, in the induction experiment of AmpC by CFZ and IMP, three clinical isolates were randomly used (isolated from blood, urine, and ascites respectively), and the induction effect of CFZ and IMP in the three strains were consistent. We only listed the results of one strain isolated from a patient with sepsis to make the manuscript more understandable. Therefore, due to the limitation of sample size, the conclusions may not apply to a very small number of *Enterobacter cloacae* complex*.*

## Conclusions

In conclusion, our study confirmed that NagZ is the key factor for CFZ and IMP to induce AmpC expression and enhance resistance in *Enterobacter cloacae* complex.Thereby providing new prospects for the treatment of multidrug-resistant *Enterobacter cloacae* complex. These prospects might include the use of NagZ inhibitors and β-lactam antibiotics to treat the infectious diseases caused by *Enterobacter cloacae* complex*.* On the other hand, CFZ and IMP must be used carefully because they might aggravate the resistance of *Enterobacter cloacae* complex.

## Methods

### Bacterial strains, plasmids, primers, and antibiotics

The comprehensive data of the types of bacterial strains, primers, and plasmids involved in the investigation are presented in Table S2, S3, and S4 of supplementary materials.

### Antibiotic susceptibility test

According to the CLSI guidelines [21], antibiotic susceptibility testing was conducted using broth microdilution and disc diffusion. *Enterobacter cloacae ATCC 13,047* and *Escherichia coli* ATCC 25,922 were employed as quality control organisms. All reagents used in antibiotic susceptibility tests were procured by Wenzhou Kangtai (Bio-kont Co. Ltd, Wenzhou, China).

### Preparation of anti-NagZ antibody

Anti-NagZ antibodies were prepared by immunizing rabbits as reported previously [34]. Briefly, molecular cloning was used to clone the EC *nagZ* coding sequence (CDS) into the pET28a vector to form the pET28a-*nagZ* vector for producing NagZ recombinant protein. The recombinant protein was isolated using Ni-NAT and identified using electrophoresis before being used to immunize New Zealand rabbits (Dashuo. Co. Ltd, Chengdu, China). Finally, the antiserum was purified by the Ni-NAT column coupled with NagZ protein. Western blotting confirmed that this antibody has an excellent specificity. The primers for amplification of *nagZ* CDS are listed in Table S4.

### Assay of AmpC β-lactamase activity

The activity of AmpC β-lactamase was investigated using the nitrocefin hydrolysis technique. The LB medium was utilized to culture the EC isolates overnight at 37 °C/250 rpm as reported earlier [35]. Sub-culturing of the overnight cultured bacterial suspension was carried out in a fresh LB milieu at a concentration of 1:100. When OD600 absorbance reaches 0.8, the organisms were collected, and 1 ml protein lysate (Shanghai Sangguang Biotechnology Co., Ltd., China) was used to suspend the bacterial pellet. The samples were lyzed through sonication using a microprobe and then centrifuged at 10,000 g for 10 min to obtain the supernatant. The protein quantification kit (Beyotime, Biotechnology, Shanghai, China) was used for the determination of protein concentration. For CFZ and IMP treatment assays, the reagents were used at the sub-culture stage. The assay of nitrocefin hydrolysis was carried out in 250 μl phosphate buffer (pH 7.0) with 50 μg/ml nitrocefin (Sigma-Aldrich; Merck-KGaA, St. Louis, Missouri, USA) and 5 μg total protein. The rate of nitrocefin hydrolysis was measured at 486 nm every 5 min at ambient temperature. The nitrocefin extinction coefficient of 20,500 M^−1^ cm^−1^ was used for the determination of AmpC- β-lactamase activity.

### RNA extraction

EC isolates were cultured as described “AmpC β-lactamase activity assay”. the RNA kit (Sangon Biotech Co. Ltd, Shanghai, China) was used for total RNA extraction following the protocol outlined by the manufacturer. NanoDropTM8000 spectrophotometer (Thermo Fisher Scientific, Waltham, Mass, USA) was employed for estimating the total RNA concentration. The total RNA was stored at -70 ℃ for determining genes expression levels. For AmpC induction assay, the SICs of CFZ and IMP were employed at the sub-culture stage.

### RT- qPCR Assays

cDNA was synthesized from 500 ng of total RNA using a FastKing gDNA Dispelling RT SuperMix kit (Tiangen Biotech Co., Ltd. Beijing, China). SuperReal PreMix Color (SYBR Green) kit (Tiangen Biotech Co., Ltd. Beijing, China) was employed for Real-time fluorescence quantitative PCR (qPCR) assay following the protocol outlined by the manufacturer. The 16S was used as an internal control in qPCR assays. The final concentration of all primers in each reaction was 0.25 μM and the amplification efficiency of all primers ranged from 91 to 96% (Table S4).

### The analysis of protein extraction and western blot

Bacterial culture and total protein preparation as described “AmpC β-lactamase activity assay” and 30 μg total protein was taken for carrying out western blot assay. For CFZ and IMP treatment assays, the reagents were employed at the sub-culture stage. Western blot analysis was carried out using the standard methodology as elaborated earlier [36]. The detailed attributes of antibodies employed in western blot assay are as follows: rabbit anti-NagZ (preparation by ourselves), mouse anti-DnaK (Abcam, Cambridge, MA, USA), rabbit anti-AmpC (Abnova Taipei, Taiwan, China), goat anti-mouse IgG-HRP and goat anti-rabbit IgG-HRP (Santa Cruz Biotechnology, Inc., Santa Cruz, CA, USA). SPOT-CCD camera was used to take images. Software image J was employed for the quantification of the intensity of protein bands and DnaK was used as the internal control.

### Construction of nagZ-knockout EC model

Using a homologous recombination method and a suicide vector, the *nagZ*-knockout EC model was built using a previously reported method [37]. Briefly, PCR was used to amplify two homologous arms DNA fragments of the *nagZ* gene. The fusion DNA fragment containing two homologous arms was procured through the fusion PCR. The fusion DNA fragment was cloned into the suicide plasmid pLP12 and identified through sequencing and PCR. The recombinant plasmid with fusion DNA fragment was then transformed into *Escherichia coli* β2163. *nagZ*-knockout EC organism was finally screened through co-culturing *Escherichia coli* β2163 with recombinant plasmid and wild-type *Enterobacter cloacae*. All the reagents and strains used in *nagZ*-knockout EC preparation were bought from Nuojing Biological Company (Knogen Biotech Co., Ltd, Guangzhou, China).

### Preparation of EC models of NagZ complementation

*nagZ* CDS was cloned into a pBAD33cm-rp4 plasmid, and authenticated by PCR and sequencing. An electroporator was then used to transform the recombinant plasmid containing *nagZ* gene (pBAD33-*nagZ*) into *Escherichia coli* β2163. Ultimately, a conjugation assay was used to transform pBAD33-*nagZ* into *Enterobacter cloacae*. *nagZ* expression of pBAD33-*nagZ* was induced by 0.05% L-Arabinose (Sangon Biotech Co. Ltd, Shanghai, China). For the antibiotic susceptibility test, L-Arabinose was initially used. While for extraction of RNA, the β-lactamase activity of AmpC, and western blot assays, L-Arabinose was applied at the stage of sub-culture. All plasmids and strains were purchased from Nuojing Biological Company.

### Statistical analysis

All data were presented as mean ± standard deviation. Statistical difference analysis between two groups was performed by GraphPad Prism5 using a Two-tailed t-test. *P* < 0.01(**) was used as statistically highly significant. Each experiment was performed at least 3 times.

## Supplementary Information

**Additional file 1:** **Fig. S1.** Western blot analysis of ampC protein expression in ECC clinical isolate treated with SICs of various antibiotics.

**Additional file 2:** **Fig. S2.** Antibiotic susceptibility tests with Kirby-Bauer method. 

**Additional file 3:** **Table S1.** MICs and SICs of various antibiotics against EC clinical isolate. 

**Additional file 4:** **Table S2.** Strains information. 

**Additional file 5:** **Table S3.** Plasmids information. 

**Additional file 6:** **Table S4.** Primers information. 

**Additional file 7.** WB raw data.

## Data Availability

Gene expression data involved in the manuscript was deposited in Gene Expression Omnibus (GEO) under accession GSE207144 (available at https://www.ncbi.nlm.nih.gov/geo/query/acc.cgi?acc=GSE207144), Sequence data (including DNA and RNA) are available from the GenBank accession mumbers (BankIt2598180 Seq1 ON892738; BankIt2598180 Seq2 ON892739; BankIt2598180 Seq3 ON892740; BankIt2598180 Seq4 ON892741).

## Abbreviations

ECC: *Enterobacter cloacae* Complex
EC: *Enterobacter cloacae*
MIC: Minimum inhibitory concentration
SIC: Subinhibitory concentrations
PIP: Piperacillin
TZP: Piperacillin-tazobactam
ATM: Aztreonam
CRO: Ceftriaxone
CAZ: Ceftazidime
SCF: Cefoperazone-sulbactam
CFZ: Cefazolin
IMP: Imipenem
Δ*nagZ*: *nagZ* *-knockout Enterobacter cloacae*
Δ*nagZ* + CFZ: Δ*nagZ* Treated with CFZ
Δ*nagZ* + IMP: Δ*nagZ* Treated with IMP
Δ*nagZ* + NagZ + CFZ: Δ*nagZ* + NagZ treated with CFZ
Δ*nagZ* + NagZ + IMP: Δ*nagZ* + NagZ treated with IMP
