# Supplementary material for: Cefazolin and imipenem enhance AmpC expression and resistance in NagZ-dependent manner in Enterobacter cloacae complex
Source: BMC Microbiol. 2022 Nov 29;22:284. doi: 10.1186/s12866-022-02707-7 (PMC9706910; doi:10.1186/s12866-022-02707-7)
Supplement: Supplementary file 1 — Additional file 1: Fig. S1. Western blot analysis of ampC protein expression in ECC clinical isolate treated with SICs of various antibiotics. [file 12866_2022_2707_MOESM1_ESM.pdf]

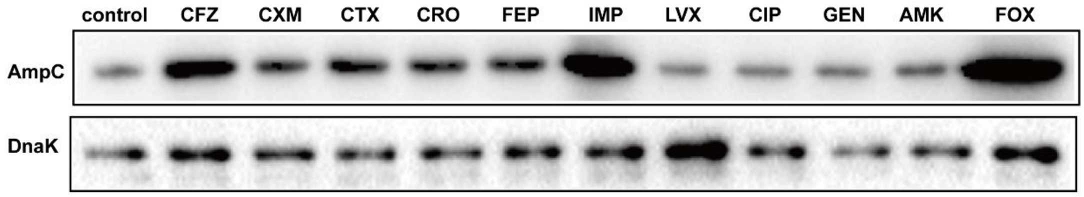

Fig. S1 Western blot analysis of *ampC* protein expression in ECC clinical isolate treated with SICs of various antibiotics. CFZ: cefazolin, CXM: Cefuroxime, CTX: cefotaxime, CRO: ceftriaxone, FEP: Cefepime, IMP: imipenem, LVX: levofloxacin, CIP: ciprofloxacin, GEN: gentamicin, AMK: Amikacin, FOX: cefoxitin (positive control).
