# Supplementary material for: Cefazolin and imipenem enhance AmpC expression and resistance in NagZ-dependent manner in Enterobacter cloacae complex
Source: BMC Microbiol. 2022 Nov 29;22:284. doi: 10.1186/s12866-022-02707-7 (PMC9706910; doi:10.1186/s12866-022-02707-7)
Supplement: Supplementary file 2 — Additional file 2: Fig. S2. Antibiotic susceptibility tests with Kirby-Bauer method. [file 12866_2022_2707_MOESM2_ESM.pdf]

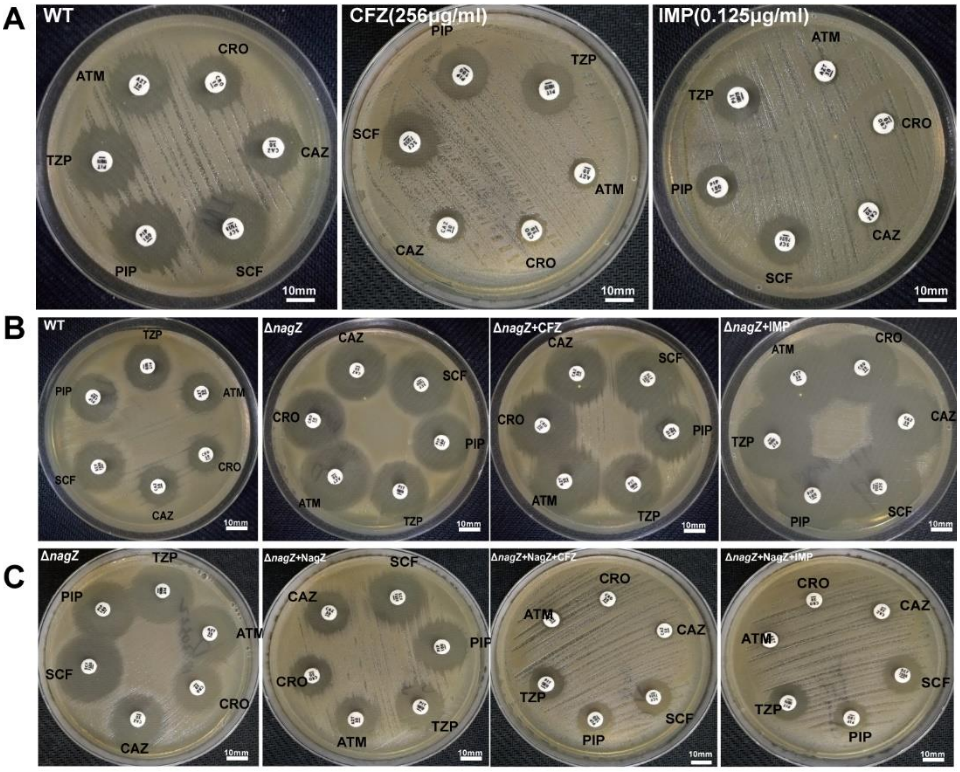

Fig. S2 Kirby-Bauer figures. (A) Antibiotic susceptibility test (Kirby-Bauer method) was employed for determining the roles of subinhibitory concentration CFZ (256μg/ml) and IMP (0.125μg/ml) in resistance in ECC clinical isolate. (B) Kirby-Bauer method was employed for determining the roles of NagZ in resistance in WT, Δ*nagZ*, Δ*nagZ*+CFZ, and Δ*nagZ*+IMP strains. (C) Inhibition zone of PIP, TZP, ATM, CRO, CAZ, and SCF was detected by Kirby-Bauer in strains of Δ*nagZ*, Δ*nagZ*+NagZ, Δ*nagZ*+NagZ+CFZ, and Δ*nagZ*+NagZ+IMP. CFZ: cefazolin, IMP: imipenem, PIP: piperacillin, TZP: piperacillin-tazobactam, ATM: aztreonam, CRO: ceftriaxone, CAZ: ceftazidime, SCF: cefoperazone-sulbactam. WT: ECC clinical isolate, Δ*nagZ: nagZ*-knockout ECC clinical isolate, Δ*nagZ*+CFZ: Δ*nagZ* treated with CFZ, Δ*nagZ*+IMP: Δ*nagZ* treated with IMP, Δ*nagZ*+NagZ: Δ*nagZ* complemented with NagZ, Δ*nagZ*+NagZ+CFZ: Δ*nagZ*+NagZ treated with CFZ, and Δ*nagZ*+NagZ+IMP: Δ*nagZ*+NagZ treated with IMP. Scale bar, 10 mm.
