# Supplementary material for: Cefazolin and imipenem enhance AmpC expression and resistance in NagZ-dependent manner in Enterobacter cloacae complex
Source: BMC Microbiol. 2022 Nov 29;22:284. doi: 10.1186/s12866-022-02707-7 (PMC9706910; doi:10.1186/s12866-022-02707-7)
Supplement: Supplementary file 3 — Additional file 3: Table S1. MICs and SICs of various antibiotics against EC clinical isolate. [file 12866_2022_2707_MOESM3_ESM.pdf]

Table S1 MICs and SICs of various antibiotics against EC clinical isolate

**Antibiotics**	**GEN**	**CRO**	**CAZ**	**LVX**	**IMP**	**AMK**	**CFZ**	**FEP**	**CTX**	**CIP**	**FOX**
MIC (μg/ml)	0.5	16	16	0.5	0.5	1	1024	0.5	16	0.5	512
SIC (μg/ml)	0.125	4	4	0.125	0.125	0.25	256	0.125	4	0.125	128
