# Supplementary material for: Cefazolin and imipenem enhance AmpC expression and resistance in NagZ-dependent manner in Enterobacter cloacae complex
Source: BMC Microbiol. 2022 Nov 29;22:284. doi: 10.1186/s12866-022-02707-7 (PMC9706910; doi:10.1186/s12866-022-02707-7)
Supplement: Supplementary file 4 — Additional file 4: Table S2. Strains information. [file 12866_2022_2707_MOESM4_ESM.pdf]

Table S2 Strains information

**strains**	**sources**	**description**
ECC clinical isolate	blood	Isolation from the blood of a diabetic patient
Enterobacter cloacae ATCC 13047	Purchase from Bio-kont Co. Ltd, Wenzhou, China	For quality control of Antibiotic susceptibility test
Escherichia coli ATCC 25922	Purchase from Bio-kont Co. Ltd, Wenzhou, China	For quality control of Antibiotic susceptibility test
Escherichia coli 21	Purchase from TransGen Biotech, Beijing, China	For NagZ recombinant protein expression
