# Supplementary material for: Cefazolin and imipenem enhance AmpC expression and resistance in NagZ-dependent manner in Enterobacter cloacae complex
Source: BMC Microbiol. 2022 Nov 29;22:284. doi: 10.1186/s12866-022-02707-7 (PMC9706910; doi:10.1186/s12866-022-02707-7)
Supplement: Supplementary file 5 — Additional file 5: Table S3. Plasmids information. [file 12866_2022_2707_MOESM5_ESM.pdf]

Table S3 Plasmids information

plasmid	description	source
pET28a	For pET28a-*nagZ*-6His vector construction	Purchase from Sangon Biotech Co.,Ltd
pET28a-*nagZ*-6His	For *nagZ* overexpression	Prepared in this study
pLP12	For *nagZ* knockout construction	Purchase from Knogen Biotech Co., Ltd
pBAD33cm-rp4(pBAD33)	For *nagZ* knockout construction and *nagZ* overexpression	Purchase from Knogen Biotech Co., Ltd
pBAD33-*nagZ*	For *nagZ* overexpression	Prepared in this study
