# Supplementary material for: Cefazolin and imipenem enhance AmpC expression and resistance in NagZ-dependent manner in Enterobacter cloacae complex
Source: BMC Microbiol. 2022 Nov 29;22:284. doi: 10.1186/s12866-022-02707-7 (PMC9706910; doi:10.1186/s12866-022-02707-7)
Supplement: Supplementary file 6 — Additional file 6: Table S4. Primers information. [file 12866_2022_2707_MOESM6_ESM.pdf]

Table S4 Primers information

Primers	Primer sequences	Amplification efficiency (%)	Product size(bp)
ampC-qPCR Forward	5' CGGATGAGGTCACGGATAAC 3'	93.3	96
ampC-qPCR Reverse	5' TGGCGTTGGCGTAAAGA 3'		
nagZ-qPCR Forward	5' ATGCGGAGGAGCGTGAAAT 3'	94.2	143
nagZ-qPCR Reverse	5' GACGGCGACCACCAGATGA 3'		
rsmA-qPCR Forward	5' AAAAGGCCAGGCGATGGTT 3'	94.7	129
rsmA-qPCR Reverse	5' GGGTGCGTTTGCAGACGAG 3'		
oxyR-qPCR Forward	5' AAGCGGGAGCGGATGAAGATA 3'	92.8	151
oxyR-qPCR Reverse	5' ATGCACGGCAGGTAAACCACA 3'		
rpoS-qPCR Forward	5' TACGATTCGCCTGCCGATTCAC 3'	91.8	254
rpoS-qPCR Reverse	5' CTTCCGGGCCGTTGTCTTTTTC 3'		
grpE-qPCR Forward	5' GCGTCGTCGTACCGAACAGG 3'	95.1	178
grpE-qPCR Reverse	5' CAGCATGGATTTCAGCGTCA 3'		
phoP-qPCR Forward	5' AGCCGATTATTATCTCAATGAACAC 3'	94.6	132
phoP-qPCR Reverse	5' GTCAGCACCAGGACAGGAAGG 3'		
16S-qPCR Forward	5' TCCTACGGGAGGCAGCAGT 3'	95.6	467
16S-qPCR Reverse	5' GGACTACCAGGGTATCTAATCCTGTT 3'		
nagZ-CDS Forward	5' ATGTTGGATGTCGAAGGGT 3'	N	1014
nagZ-CDS Reverse	5' TTAAAGGGCTGCTTTATGTG 3'		

qPCR: Real-time fluorescence quantitative polymerase chain reaction, CDS: coding sequence. N: no detection.
