# Supplementary material for: Cefazolin and imipenem enhance AmpC expression and resistance in NagZ-dependent manner in Enterobacter cloacae complex
Source: BMC Microbiol. 2022 Nov 29;22:284. doi: 10.1186/s12866-022-02707-7 (PMC9706910; doi:10.1186/s12866-022-02707-7)
Supplement: Supplementary file 7 — Additional file 7. WB raw data. [file 12866_2022_2707_MOESM7_ESM.pdf]

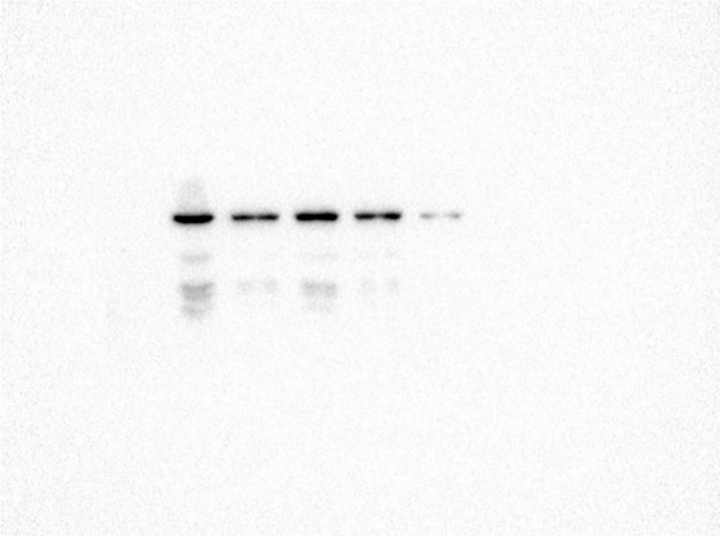

figure 1B AmpC

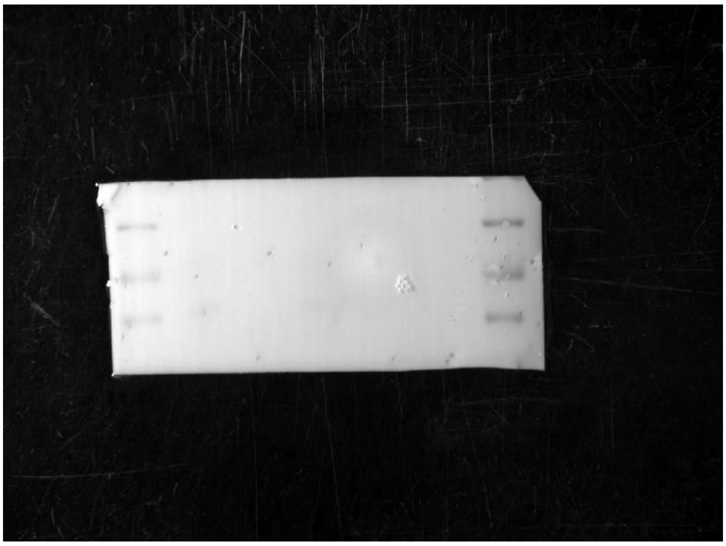

figure 1B AmpC membrane

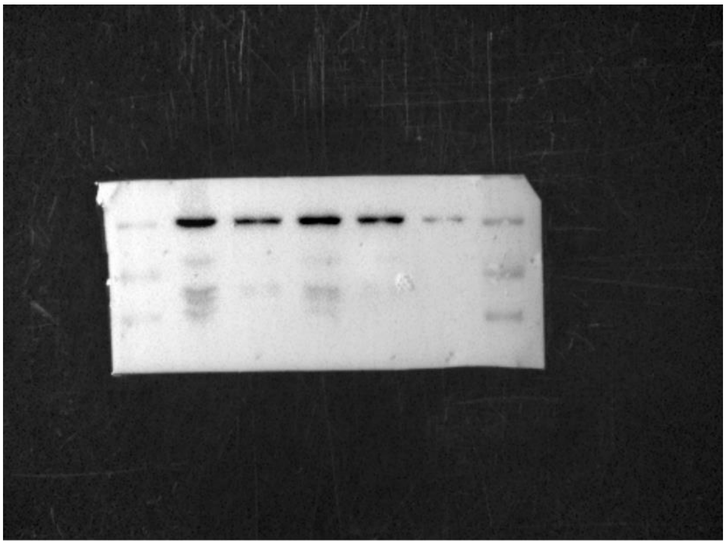

figure 1B AmpC merge

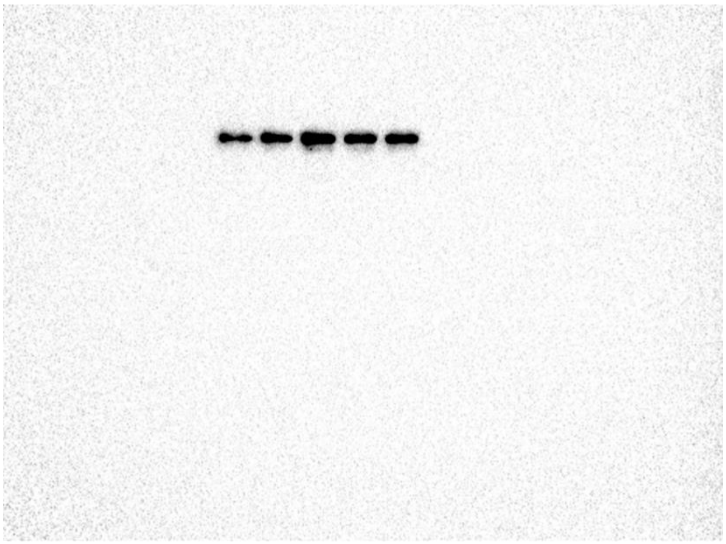

figure 1B DnaK

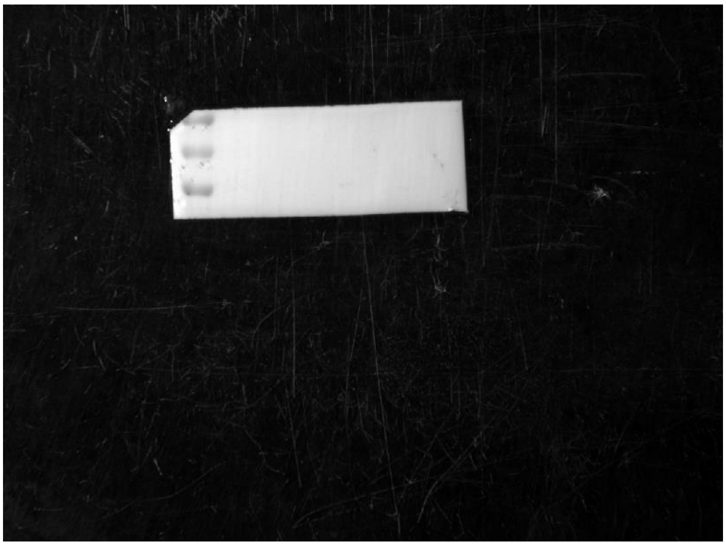

figure 1B DnaK membrane

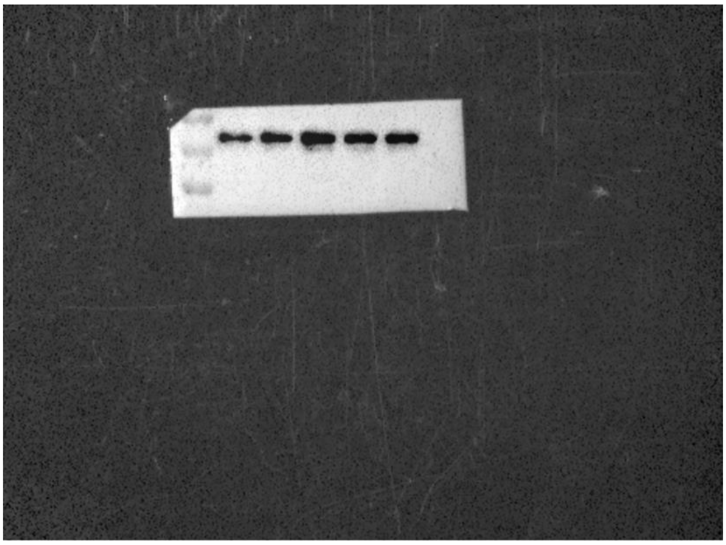

figure 1B DnaK merge

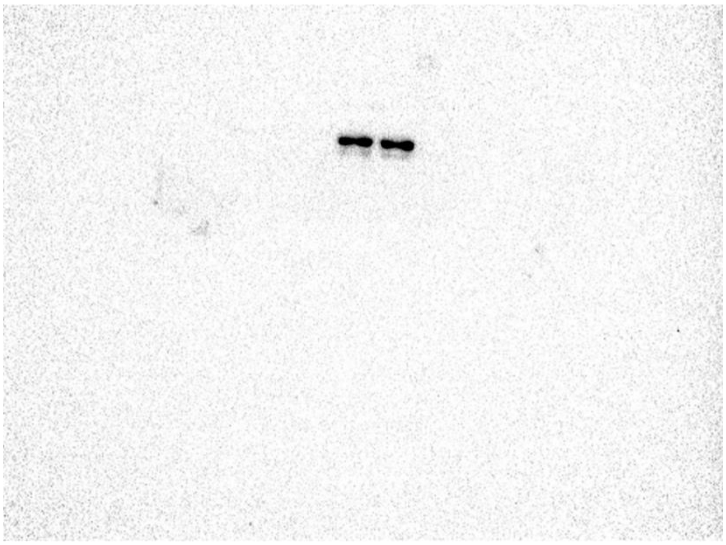
figure 2B DnaK

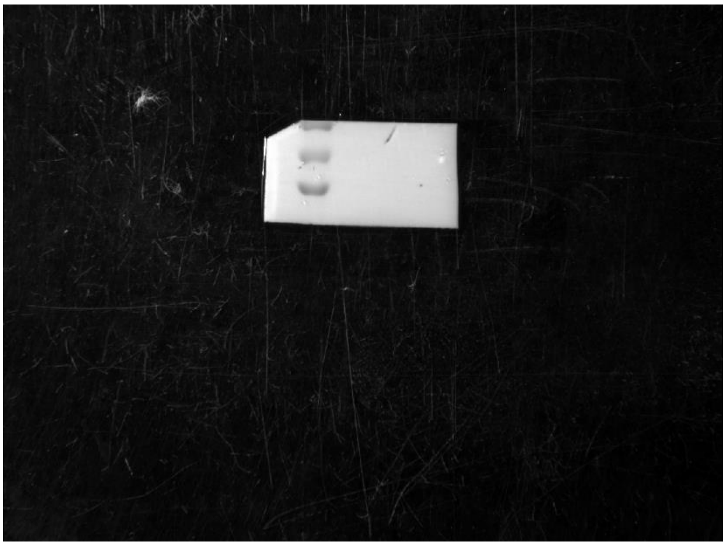
figure 2B DnaK membrane

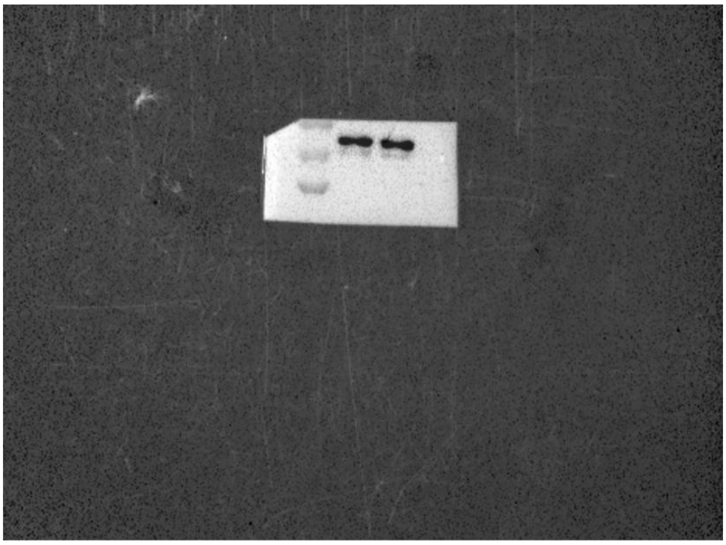
figure 2B DnaK merge

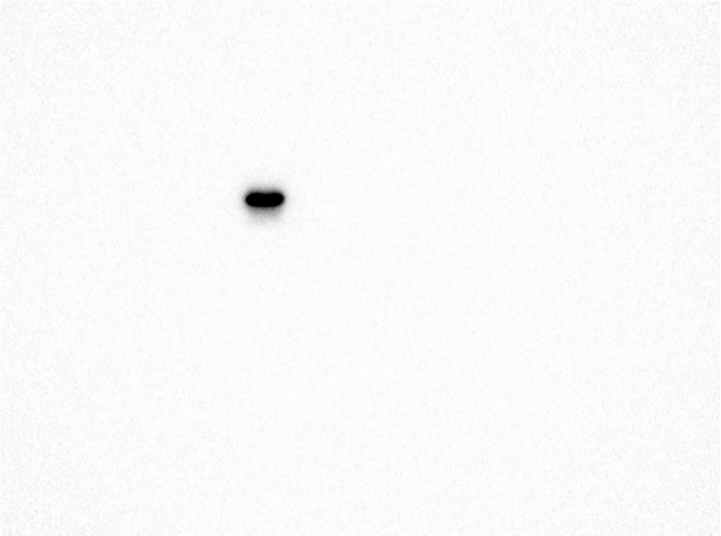
figure 2B NagZ

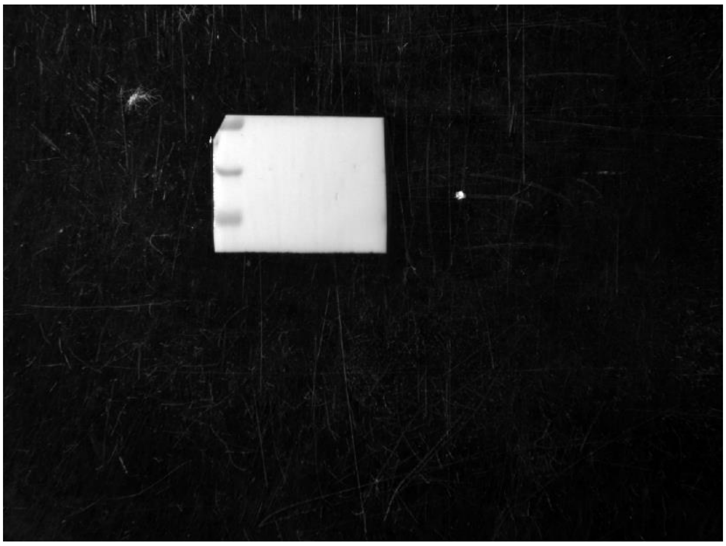
figure 2B NagZ membrane

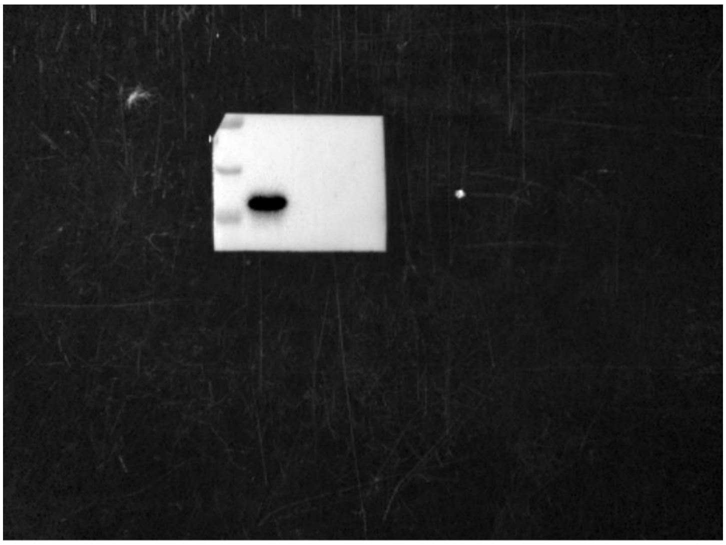
figure 2B NagZ merge

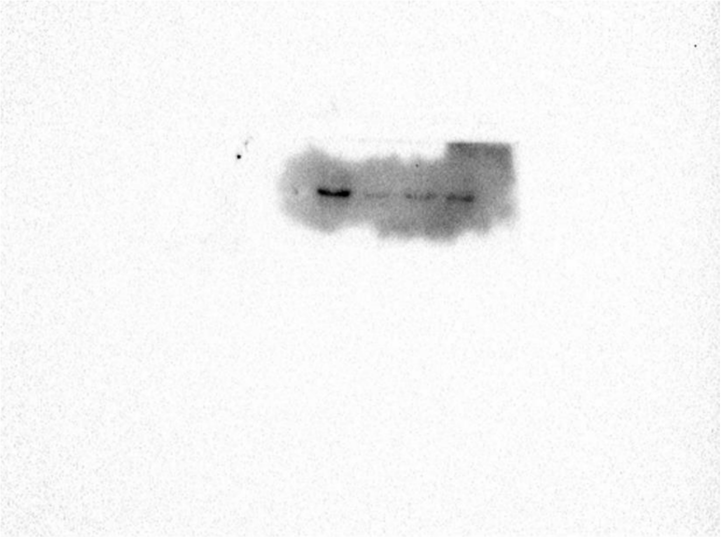

figure 2D AmpC

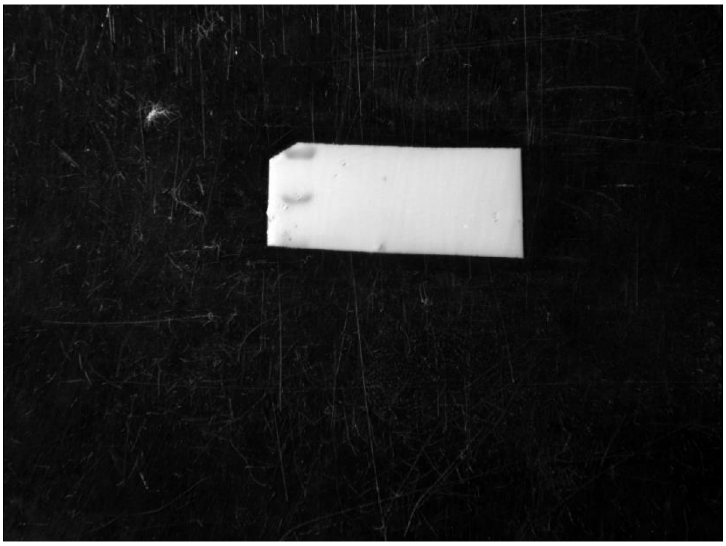

figure 2D AmpCmembrane

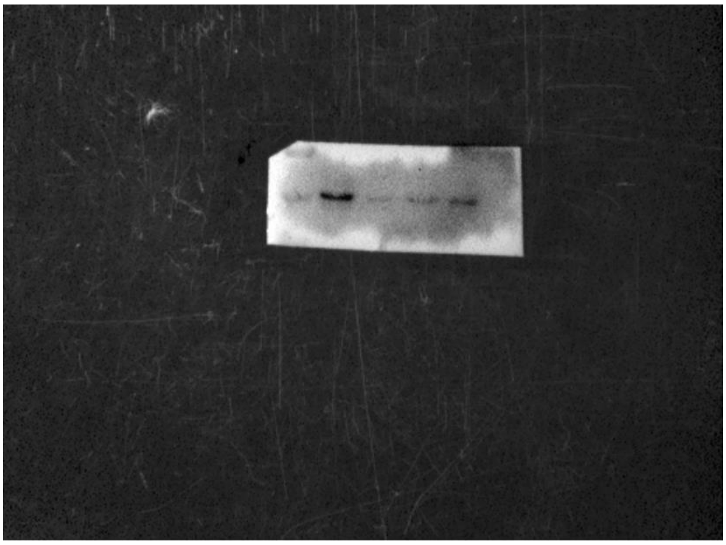

figure 2D AmpC merge

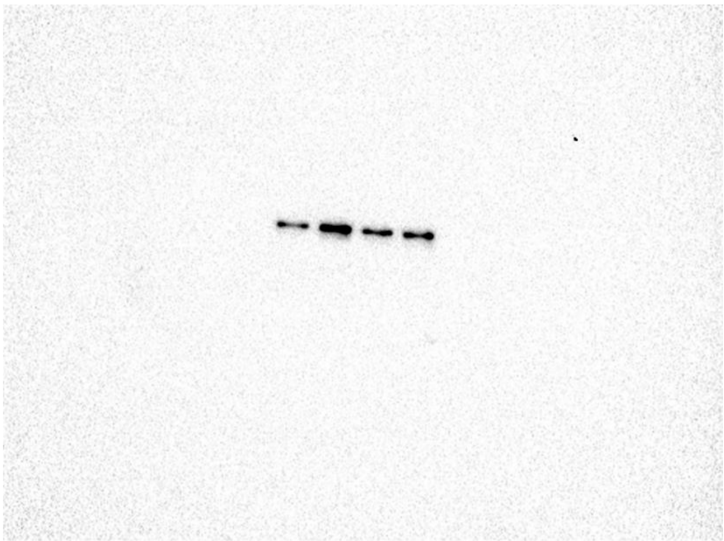

figure 2D DnaK

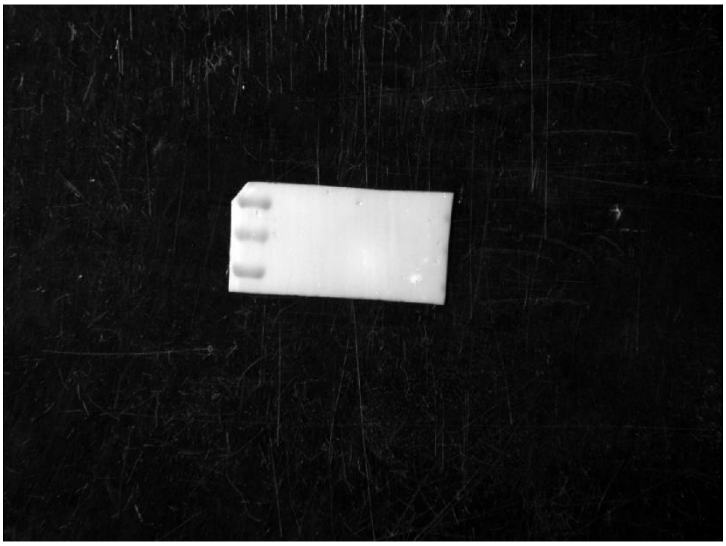

figure 2D DnaK membrane

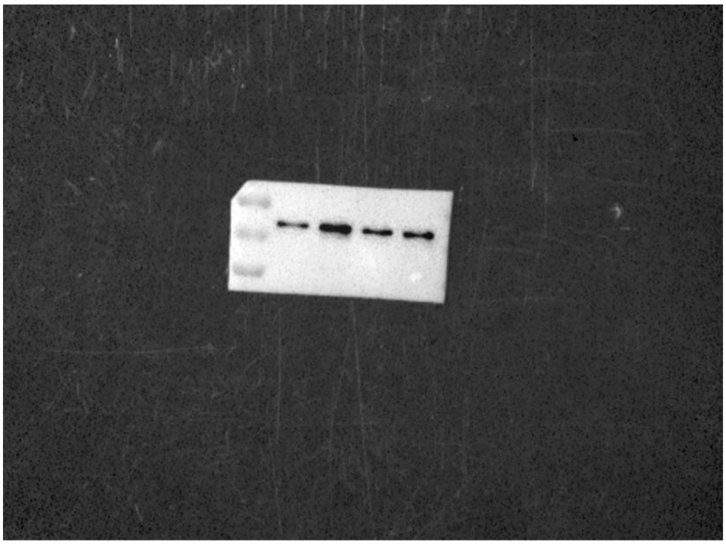

figure 2D DnaK merge

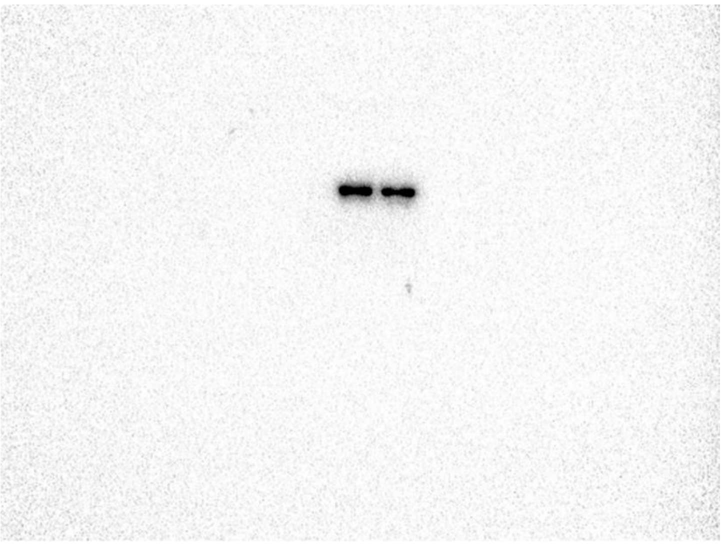

figure 3B DnaK

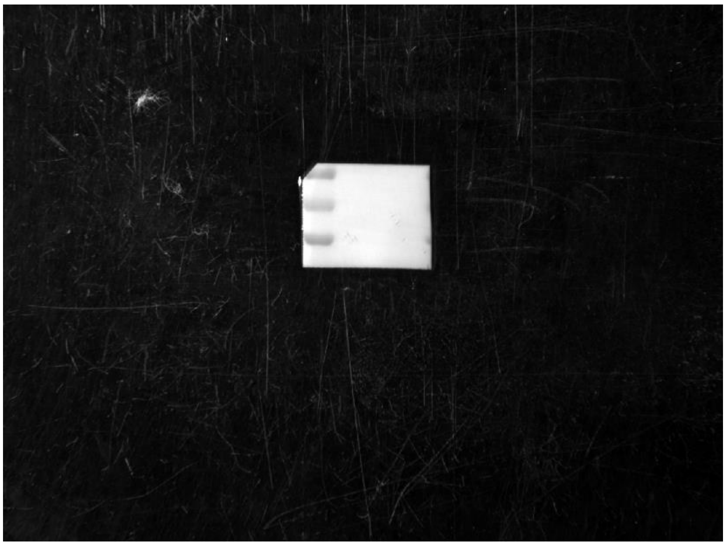

figure 3B DnaK membrane

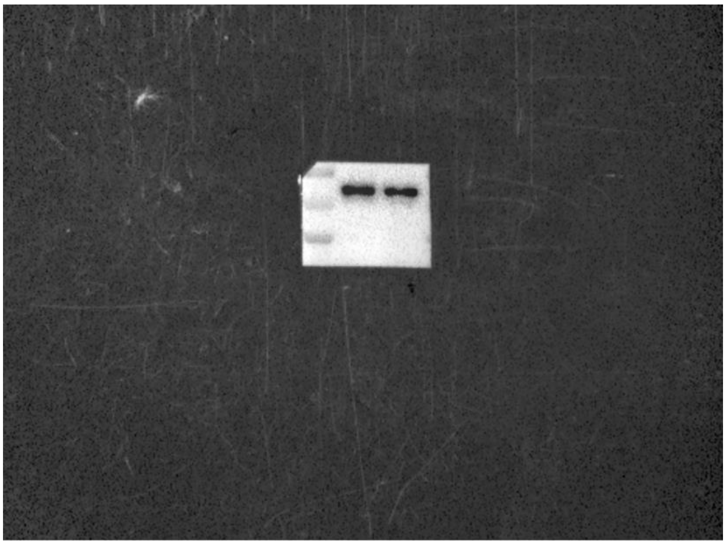

figure 3B DnaK merge

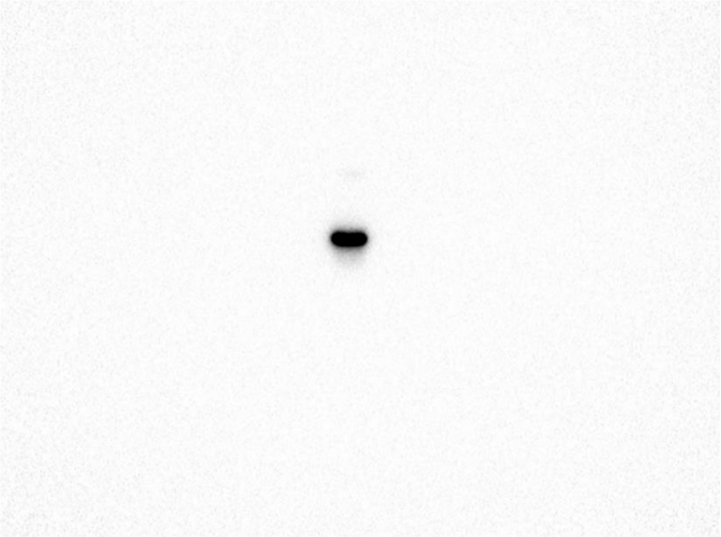

figure 3B NagZ

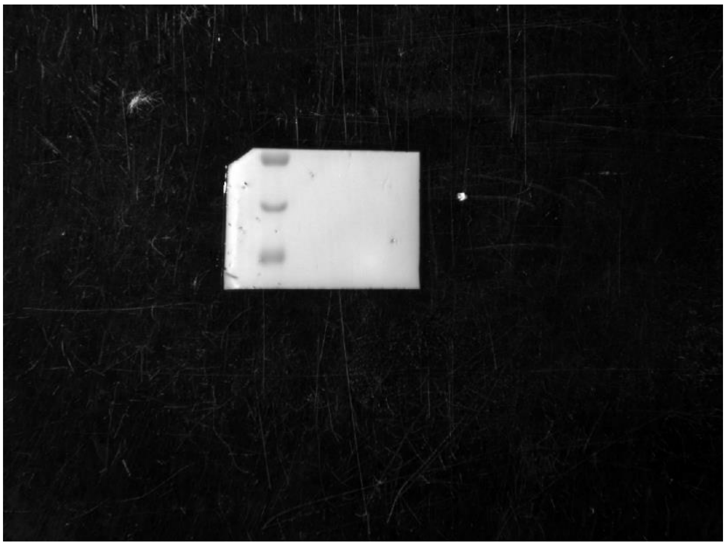

figure 3B NagZ membrane

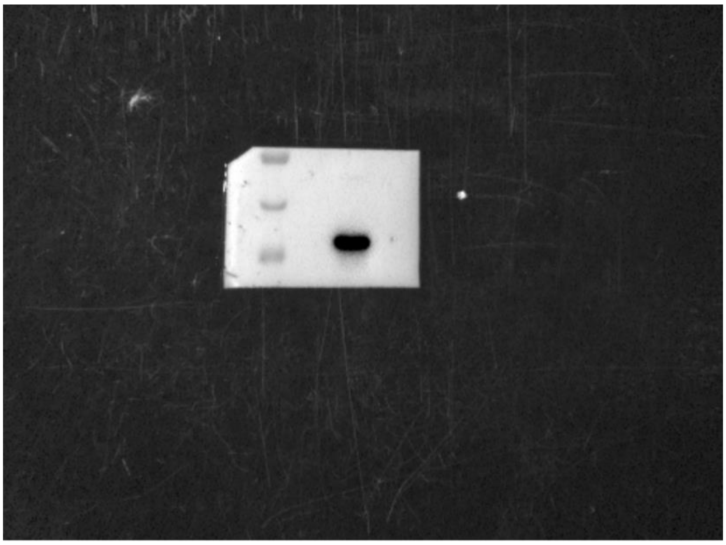

figure 3B NagZ merge

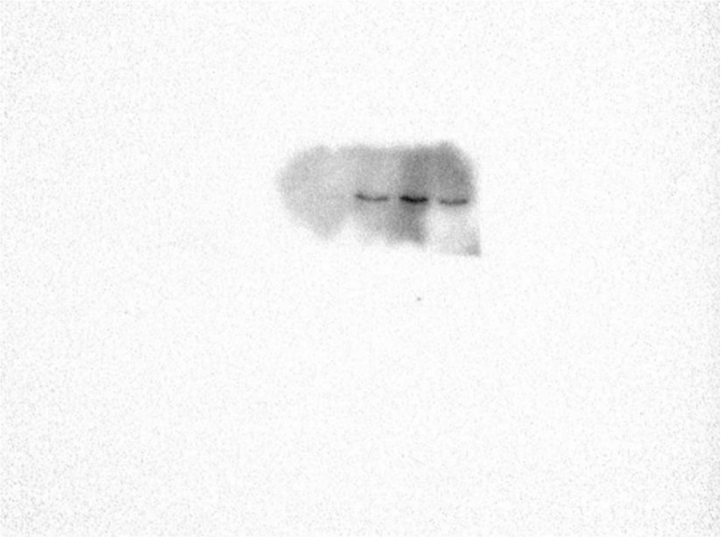
figure 3D AmpC

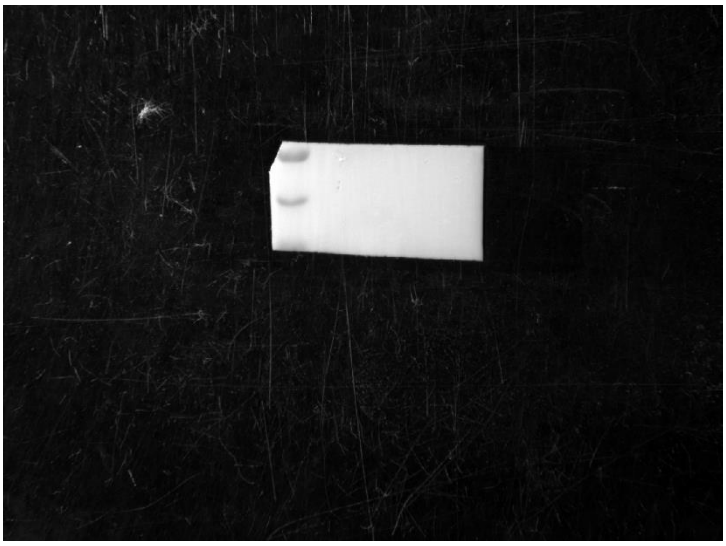
figure 3D AmpCmembrane

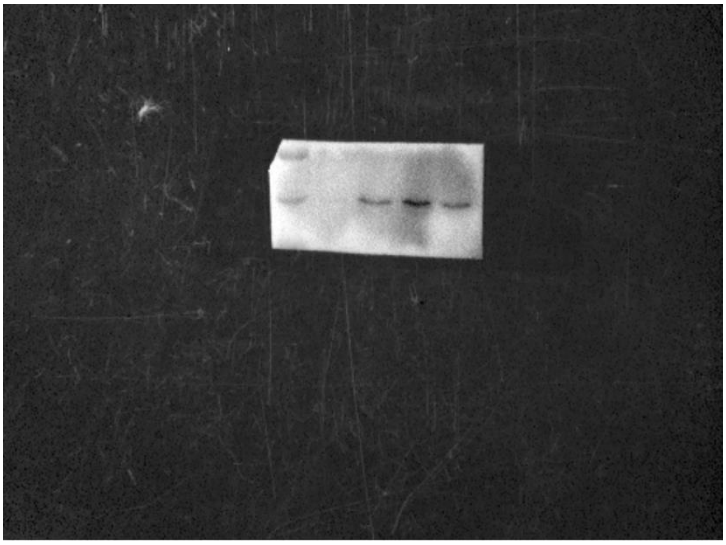
figure 3D AmpC merge

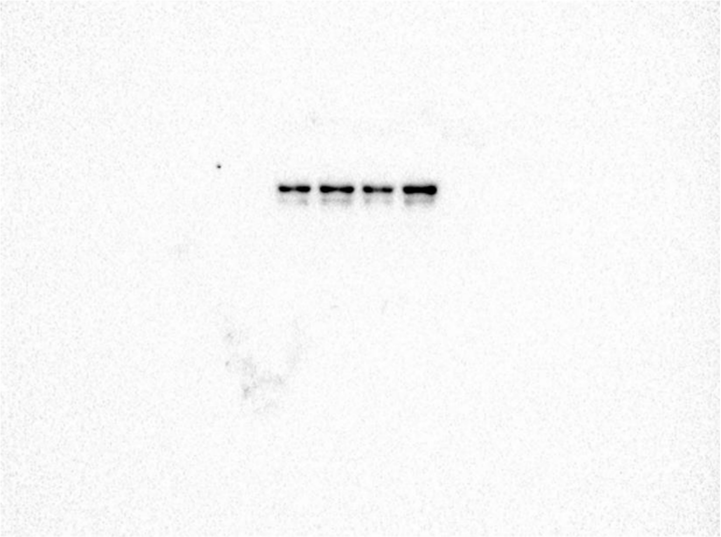

figure 3D DnaK

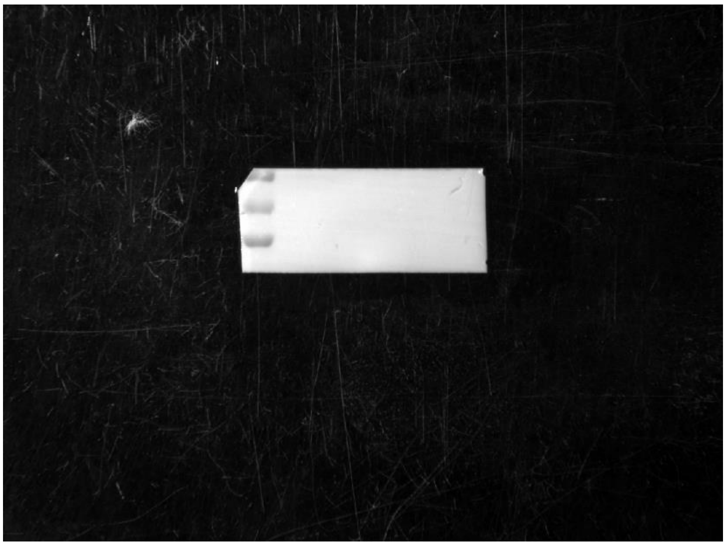

figure 3D DnaK membrane

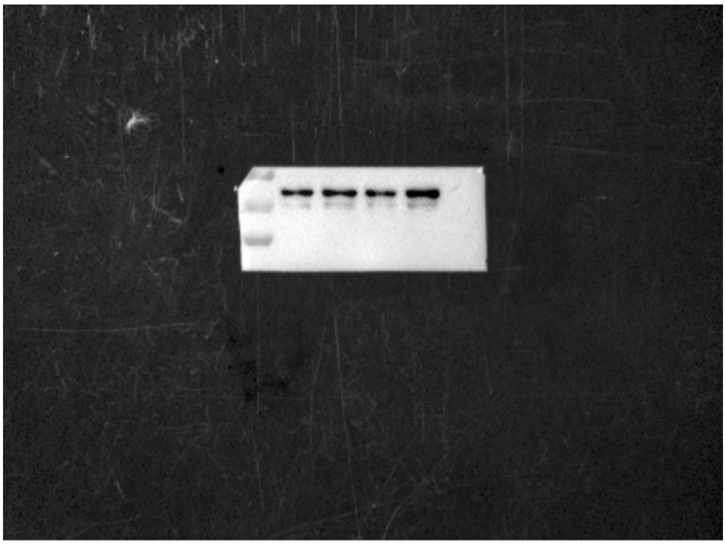

figure 3D DnaK merge

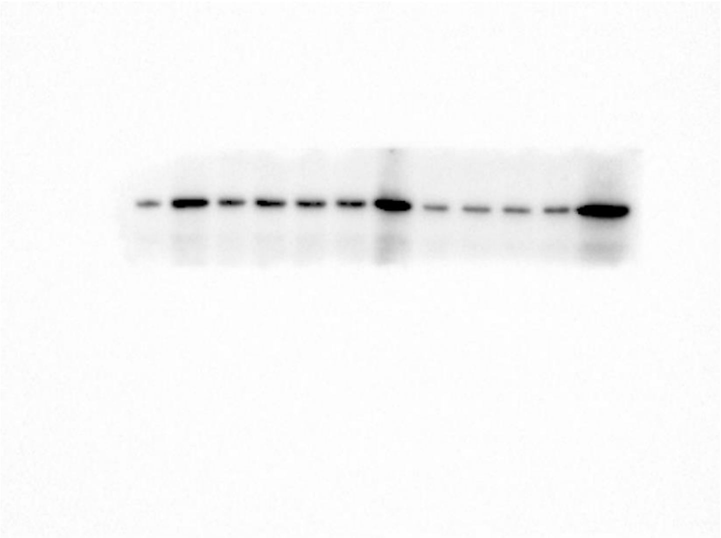

figure S1 AmpC

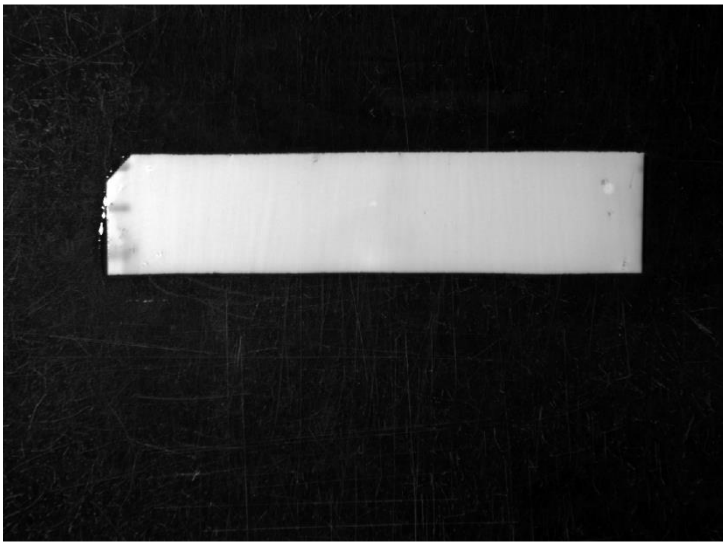

figure S1 AmpC membrane

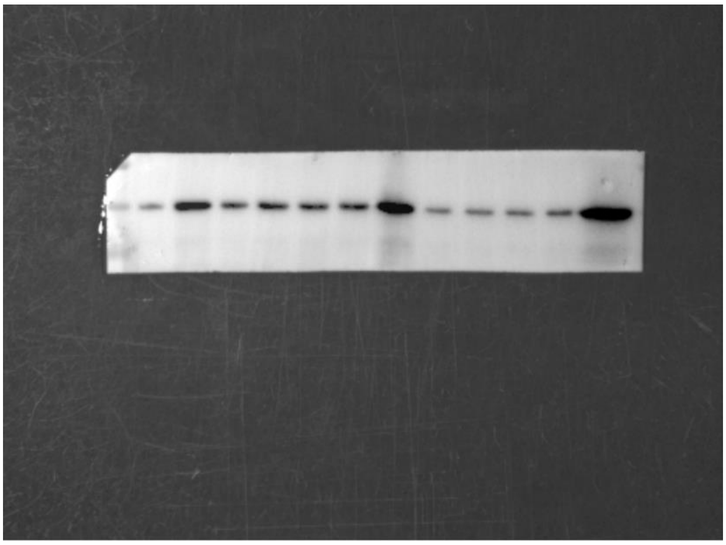

figure S1 AmpC merge

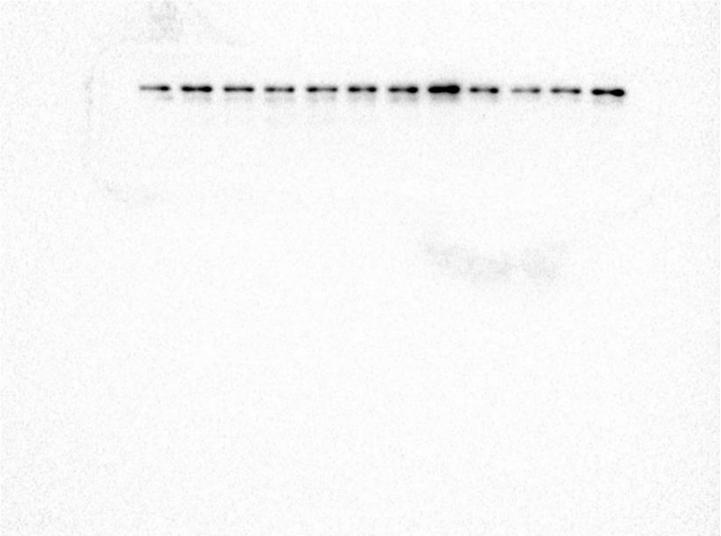

figure S1 DnaK

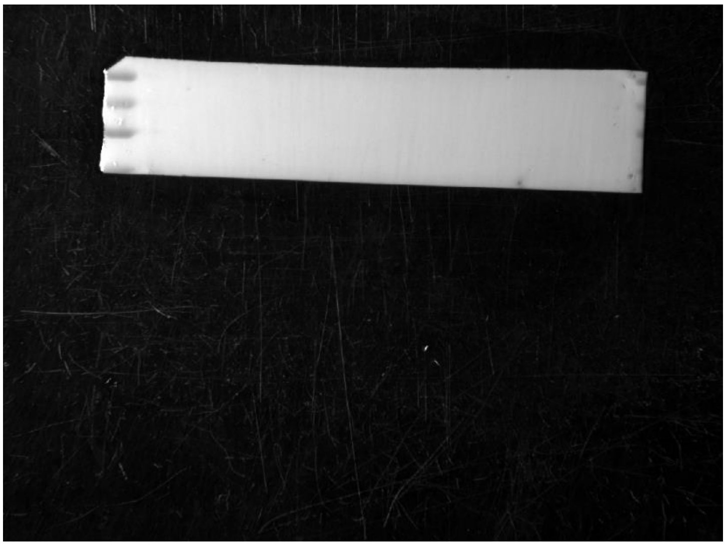

figure S1 DnaK membrane

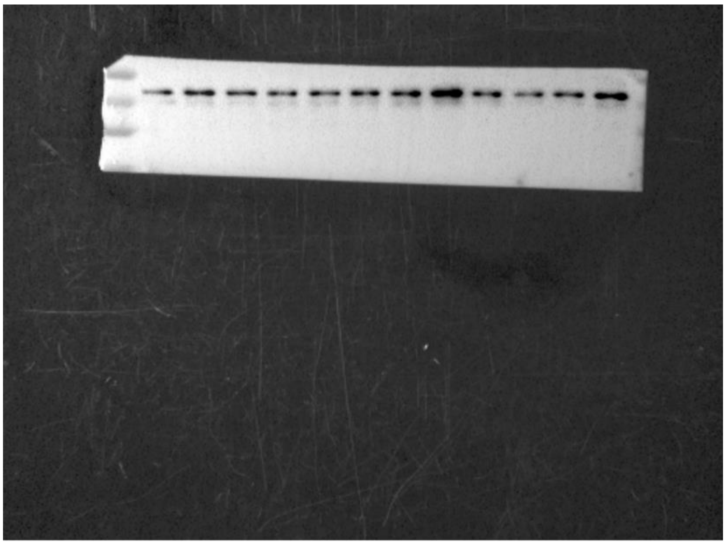

figure S1 DnaK merge
